# TCM Zheng Classification and Clinical Trials

**DOI:** 10.1155/2013/723659

**Published:** 2013-08-22

**Authors:** Aiping Lu, Alan Bensoussan, Jianping Liu, Zhaoxiang Bian, William C. S. Cho

**Affiliations:** ^1^Institute of Basic Research in Clinical Medicine, China Academy of Chinese Medical Sciences, Beijing 100700, China; ^2^School of Chinese Medicine, Hong Kong Baptist University, Kowloon 00852, Hong Kong; ^3^Centre for Complementary Medicine Research (CompleMED), University of Western Sydney, Penrith, NSW 2751, Australia; ^4^Center for Evidence Based Chinese Medicine, Beijing University of Chinese Medicine, Beijing 100029, China; ^5^Queen Elizabeth Hospital, Kowloon 00852, Hong Kong

According to the traditional Chinese medicine (TCM) theory, TCM Zheng (or pattern, syndrome) is an analysis on disease presentation by TCM practitioners. Zheng classification (*Bian Zheng*) is a traditional diagnostic method to categorize patients' syndromes based on their presented conditions. Currently, combination of Zheng classification and biomedical diagnosis becomes a common model in TCM diagnostics in clinical practice. Clinical treatments of a patient rely on the classification of a specific TCM Zheng. This special issue collects articles describing the clinical trials with TCM Zheng classification, Zheng classification methodology from clinical data, systematic reviews on clinical studies using Zheng classification, and the clinical pharmacological evaluation on TCM interventions using the omics platforms as well as bioinformatics.

Thirty-two papers are published in this special issue. Six are randomized controlled trials, one is longitudinal study, three narrative reviews, and three systematic reviews. Another five studies deal with the validity and reliability of a new Zheng diagnostic tool. In the five research articles, metabolomic analyses reveal profound differences in Zheng classification. Eight pieces of work explore molecular mechanisms of Zheng. Finally, one detects the relative temperature of different Zheng classifications with the infrared thermal imaging technology in the trial.

Among included randomized controlled trials, *“Improving the efficacy of conventional therapy by adding andrographolide sulfonate in the treatment of severe hand, foot, and mouth disease: a randomized controlled trial”* by Li et al. first provides evidence on andrographolide sulfonate for its additional effect for the treatment of severe hand, foot, and mouth disease. *“Effect of combining therapy with traditional Chinese medicine-based psychotherapy and herbal medicines in women with menopausal syndrome: a randomized controlled clinical trial”* by Yang et al. addresses the effectiveness of combined TCM-based psychotherapy and Chinese herbal medicine in the treatment of menopausal syndrome. *“Efficacy of Chuanxiong Ding Tong herbal formula granule in the treatment and prophylactic of migraine patients: a randomized, double-blind, multicenter, placebo-controlled trial”* by Fu et al. evaluates the efficacy of traditional Chinese herbal Chuanxiong Ding Tong herbal formula granule for migraine patients with the syndrome of liver wind and blood stasis. *“The effects of Xuefu Zhuyu and Shengmai on the evolution of syndromes and inflammatory markers in patients with unstable angina pectoris after percutaneous coronary intervention: a randomised controlled clinical trial”* by Wang explores six syndrome factors and four inflammatory markers in 90 participants. *“Chinese herbal medicine (Zi Shen Qing) for mild-to-moderate systematic lupus erythematosus: a pilot prospective, single-blinded, randomized controlled study”* by Zhong et al. showed that Zi Shen Qing is safe and effective for decreasing severity of systemic lupus erythematosus activity and withdrawal dosage of corticosteroids in the mild-to-moderate patients with *“*Deficiency of Qi and Yin*”* Pattern. *“Therapeutic efficacy of Fuzheng-Huayu tablet based traditional Chinese medicine syndrome differentiation on hepatitis-B-caused cirrhosis: a multicenter double-blind randomized controlled trail”* by Song et al. revealed that Fuzheng-Huayu decreases TCM syndrome scores and improves the quality of life of hepatitis-B-caused cirrhosis patients. The only longitudinal study included in this issue, *“Increases in Xu Zheng and Yu Zheng among patients with breast cancer receiving different anticancer drug therapies”* by Huang et al. assessed Zheng using the TCM Constitutional Scale. It is significant that more and more clinical trials pay attention to the Zheng classification and put it as a criterion for participants selection and outcome assessment, and such changes not only provide the change to demonstrate the characteristics of TCM therapy, but also provide the more comprehensive assessment of an intervention compared with conventional biomarker-oriented assessment.

Reviews and evidences, *“Prescription of Chinese herbal medicine and selection of acupoints in pattern-based traditional Chinese medicine treatment for insomnia: a systematic review”* by Yeung et al. included 227 studies, 17,916 subjects, and 87 TCM patterns. They highlighted high-quality studies to examine the additional benefits of pattern differentiation and pattern-based TCM treatment. *“Modified Dachengqi decoction combined with conventional treatment for treating acute exacerbation of chronic obstructive pulmonary disease: a systematic review based on randomized controlled trials”* by Wu et al. analyzed 16 studies involving 1,112 patients and showed that Dachengqi decoction combined with routine treatment enhances the effectiveness. *“Tianma Gouteng Yin as adjunctive treatment for essential hypertension: a systematic review of randomized controlled trials”* by Wang et al. reveals no confirmed conclusion about the effectiveness and safety of Tianma Gouteng Yin for essential hypertension. *“ZHENG-omics application in ZHENG classification and treatment: Chinese personalized medicine”* by Dai et al. gives the brief introduction and preliminary validation and discusses strategies and system-oriented technology. *“Clinical applications of omics technologies on ZHENG differentiation research in traditional Chinese medicine”* by Song summarized the typical omics application in common studied diseases and TCM Zhengs and discussed the main problems and countermeasure of Zheng classification researches. *“Syndrome differentiation in Chinese herbal medicine for irritable bowel syndrome: a literature review of randomized trials”* by Li et al. involved 735 RCTs and 67,784 patients and indicated that 30.5% studies applied Zheng classification. 

*“Metabonomics-based study of clinical urine samples in suboptimal health with different syndromes”* by Cui et al. identified the specific metabolic products of the syndrome of stagnation of liver Qi. *“Similar connotation in chronic hepatitis B and nonalcoholic fatty liver patients with dampness-heat syndrome”* by Dai et al. discussed the molecular mechanism of similar connotation that exists in chronic hepatitis B and nonalcoholic fatty liver by metabonomics to give the modern understanding of dampness-heat syndrome. *“Differences of excess and deficiency Zheng in patients with chronic hepatitis B by urinary metabonomics”* by Sun et al. suggested that chronic hepatitis B patients could be categorized into two groups with diverse pathogenesis. *“Differences in metabolites of different tongue coatings in patients with chronic hepatitis B”* by Zhao demonstrated that tongue coatings have objective material basis. *“Metabonomics study of essential hypertension and its Chinese medicine subtypes by using gas chromatography-mass spectrometry and nuclear magnetic resonance spectroscopy”* by Li et al. distinctly classified Yin deficiency, Yang-hyperactivity syndrome, and Yin-Yang deficiency syndrome by principal components analysis and partial least squares discriminant analysis. 

Zheng diagnosis for patients is sometimes inconsistent among different practitioners. This is in part due to the paucity of evidence-based diagnostic methods in TCM. To solve this problem, establishment of validated diagnostic tool is inevitable. *“Visual agreement analyses of traditional Chinese medicine: a multiple-dimensional scaling approach”* by Lo et al. developed a novel visual agreement analysis for TCM via multiple-dimensional scaling. *“The pattern element scale: a brief tool of traditional medical subtyping for dementia”* by Shi et al. evaluated the utility of the new pattern element scale in dementia patients. *“Validity of a diagnostic scale for acupuncture: application of the item response theory to the five viscera score”* by Tomura first applied the item response theory model to the five viscera score to test its validity by evaluating the ability of the questionnaire items to identify an individual's latent traits. *“Interrater reliability of Chinese medicine diagnosis in people with prediabetes”* by Grant et al. provides initial evidence of variation in the biomarkers of people with prediabetes according to the different TCM Zhengs. *“Validation of the constitution in Chinese medicine questionnaire: does the traditional Chinese medicine concept of body constitution exist?”* by Wong et al. adapted and validated the constitution in Chinese medicine questionnaire in Hong Kong Chinese people. *“Classification and progression based on CFS-GA and C5.0 boost decision tree of TCM Zheng in chronic hepatitis B”* by Chen et al. applied correlation-based feature selection, genetic algorithm, and decision tree for Zheng classification and progression in the TCM treatment of chronic hepatitis B (CHB). *“Understanding the molecular mechanism of interventions in treating rheumatoid arthritis patients with corresponding traditional Chinese medicine patterns based on bioinformatics approach*” by Jiang et al. indicates that molecular network analysis could provide insight into the full understanding of the underlying mechanism of how TCM pattern impacts the efficacy. *“The correlation between high-sensitivity C-reactive protein, matrix metallopeptidase 9, and traditional Chinese medicine syndrome in patients with hypertension”* by Wu et al. investigated the relationships between Zheng and the two inflammatory biomarkers in patients with essential hypertension. *“Research on Zheng classification fusing pulse parameters in coronary heart disease”* by Guo et al. illustrated that the nonlinear dynamic variables of TCM pulse can improve the performances of Zheng classification models. *“A two-level model for the analysis of syndrome of acute ischemic stroke: from diagnostic model to molecular mechanism”* by Dai et al. analyzed that collateral obstruction syndrome, the integration of diagnostic model and molecular mechanism analysis creates an interesting perspective to better understand Zheng. *“Progression from excessive to deficient syndromes in chronic hepatitis B: a dynamical network analysis of miRNA array dat*a*”* by Chen et al. suggested that miRNAs are important mediators for TCM Zheng classification and could be potential diagnosis and therapeutic molecular markers. *“Circulating miR-583 and miR-663 refer to ZHENG differentiation in chronic hepatitis B”* by Zhang et al. indicated that 354 putative targets for miR-583 and 68 putative targets for miR-663 were mainly involved in Axon guidance, Neurotrophin, and MAPK signaling pathway; miR-583 and miR-663 may be potential markers for Zheng differentiation in CHB. Besides, *“Autophagy inhibition enhances apoptosis induced by dioscin in Huh7 cells”* by Hsieh et al. showed that dioscin, a new water-soluble steroidal saponin from Dioscorea nipponica Makino, causes autophagy in Huh7 cells and suggested that dioscin has a cytoprotective effect.

Furthermore, *“Clinical trial on the characteristics of Zheng classification of pulmonary diseases based on infrared thermal imaging technology”* by Ni et al. showed that the infrared thermal images characteristics of a different Zheng of pulmonary disease were distinctly different. These studies provide the potential of how to improve the quality of Zheng diagnosis. Further studiese are needed to systematically improve the quality of Zheng diagnosis and the integrate this method with modern medicine.

Recently, a great progress has been made in the clinical studies focusing on the exploration of the effect of TCM interventions in the patients with clarified TCM Zheng in some diseases. The randomized controlled trials and systematic reviews provide strong evidence for Zheng classification. This special issue presents, even though partially, the efficacy of TCM practice with integration of TCM Zheng classification, biomedical disease diagnosis, and the biological basis of Zheng. We hope that our readers could share these works and further contribute to this important field.

